# Effect of Aerobic Exercises on Serum Levels of Apolipoprotein A1 and Apolipoprotein B, and Their Ratio in Patients with Chronic Obstructive Pulmonary Disease

**Published:** 2018-02

**Authors:** Rostam Yazdani, Hamid Marefati, Armita Shahesmaeili, Samira Nakhaei, Alireza Bagheri, Maryam Dastoorpoor

**Affiliations:** 1 Internal Medicine Department, Kerman University of Medical Sciences, Kerman, Iran; 2 Department of Sport Sciences, Faculty of Human Sciences, Bojnord University, Bojnord, Iran; 3 Cardiovascular Research Center, Institute of Basic and Clinical Physiology Sciences, Kerman University of Medical Sciences, Kerman, Iran; 4 Gastroenterology and Hepatology Research Center, Kerman University of Medical Sciences, Kerman Iran; 5 Internal Medicine Department, Kerman University of Medical Sciences, Kerman, Iran; 6 Department of Physical Education and Sport Science, Shahid Bahonar University Kerman, Iran; 7 Air Pollution and Respiratory Diseases Research Center, Ahvaz Jundishapur University of Medical Sciences, Ahvaz, Iran.

**Keywords:** Aerobic Exercises, Apolipoprotein A1, Apolipoprotein B, Chronic Obstructive

## Abstract

**Background:**

Cardiovascular disease is one of the most common disorders associated with chronic obstructive pulmonary disease (COPD). There are few studies on the effects of physical exercises, especially aerobic exercises, on serum levels of apolipoprotein A1 and apolipoprotein B in patients with COPD. The current study aimed at determining the effect of aerobic exercises on serum levels of apolipoprotein A1 and B and apo-A1/apo-B ratio.

**Materials and Methods:**

In the current randomized, controlled, clinical trial, with a pretest posttest control group design, 22 males with COPD were randomly assigned to the aerobic exercise and control groups. The aerobic exercise program was performed within two months based on three 30–40-minute sessions per week. Serum levels were measured and evaluated before and after aerobic exercises. Data were analyzed using the paired samples *t* test.

**Results:**

In the aerobic exercise group, the mean of Apo A1 and Apo B after the intervention (169.36±5.42 and 93.63±5.24 mg/dL, respectively) was significantly higher than that of before the intervention (146±6.09 and 83.27±4.44 mg/dL, respectively) (P-value=0.001). However, apoA1/Apo B ratio did not significantly change after the intervention compared with that of before the intervention (1.85±0.10 vs. 1.80±0.13) (P >0.05). There was no significant change in the mean Apo A1 and Apo B levels and Apo A1/Apo B ratio after the intervention in the control group.

**Conclusion:**

Regular aerobic physical exercises are effective in increasing the serum level of Apo A1 in patients with COPD. Due to the proven protective role of Apo A1 in patients with COPD, this biomarker can improve respiratory efficacy in such patients.

## INTRODUCTION

Apolipoprotein A1 (Apo A1) reduces pulmonary oxidative stress, inflammation, and collagen sedimentation (1); serum Apo B leads to the production of a large number of atherogenic particles (2). A large number of studies showed that the elevated level of Apo B and a reduction in Apo A1/Apo B ratio are more valuable than other cholesterol parameters to predict the risk of cardiovascular diseases (3–12). The effect of aerobic exercises on Apo A1 and Apo B levels was evaluated in some studies and the results showed that aerobic exercises can affect the levels of such parameters, increase Apo A1/Apo B ratio, and effectively reduce the risk factors of cardiovascular diseases (13).

The increasing prevalence of compulsive obstructive pulmonary disease (COPD) as one of the priorities of the World Health Organization (WHO) has a significant impact on the health care systems, since it was the third leading cause of death by 2020 (14). According to the WHO, 80 million people have COPD worldwide, of which 12 million living in the United States(15). It poses many social and economic burdens to the community including direct costs, medical care costs, and indirect costs including costs of absenteeism, occupancy of hospital beds, and medical staff (16).

Chronic inflammation, oxidative stress, and proteolysis play a major role in the pathogenesis of COPD and emphysema(14). COPD has significant extrapulmonary effects associated with its intensity, such as cardiovascular diseases, diabetes mellitus, renal failure, osteoporosis, and psychiatric disorders, which can have a negative impact on patients’ quality of life. The long-term survival of the disease is associated with its intensity and the developing disorders(16). Cardiovascular diseases are the most common complications associated with COPD, which lead to increased mortality in such patients (17–20). It seems that COPD benefits from various mechanisms such as oxidative stress(21) and direct effect of vascular remodeling on cardiac function(22).

Apo A1, which is only produced by the liver and intestine, is a structural protein of high-density lipoproteins (HDL) (23). It is observed that the Apo A1 emulates the synthetic peptides in animal models and has a protective effect on acute pulmonary damage, asthma, pulmonary hypertension, influenza pneumonia, and emphysema; this feature can be used for new therapies. Furthermore, it decreases the risk of lung neoplasia and the trend of lung tumors growth (24).

Apolipoprotein B in the liver is bound to the LDL and VLDL precursors. Apolipoprotein B is the only protein in the LDL, and is also a component of VLDL, Lipoprotein A, and metabolic residues of VLDL and chylomicrons (25, 26).

Considering the extensive researches on identifying the factors affecting the development and progression of COPD, and the need for new methods for the better management of this disease and its associated disorders, which cardiovascular diseases are of the most important ones, the current study aimed at determining the effect of aerobic exercises on the serum levels of Apo A1 and Apo B, their ratio, and their roles in improving lung efficacy and reducing the risk of cardiovascular diseases in patients with COPD.

## MATERIALS AND METHODS

In the current randomized, controlled, clinical trial with pretest and posttest control group design, conducted in Kerman, Iran, 22 patients with COPD were selected by the random sampling method. The study was also registered in the Iranian Registry of Clinical Trials (IRCT) under the code, IRCT20171224038032N1. The Ethics Committee of Kerman University of Medical Sciences, Kerman, Iran, approved the study protocol.

According to the results of the previous study and considering α = 5% and β = 10% Mean1 = 23.71, Mean = −17.35, sd1 = 12.49, sd2 = 11.29 (27), and based on the formula of comparison of the means, a sample size of 2 people was calculated in each intervention and control groups. Considering the difference in our study with this study, in order to increase the accuracy of the results, the final sample size was considered to be 11 in each intervention and control group.

$$\text{n}=\frac{{\left({\text{Z}}_{\left(1-\alpha /2\right)}+{\text{Z}}_{\left(1-\beta \right)}\right)}^{2}\left({{\text{sd}}_{1}}^{2}+{{\text{sd}}_{2}}^{2}\right)}{{\text{d}}^{2}}$$

Inclusion criteria of the current study were: age range 40–70 years and body mass index (BMI) of 19 to 24 kg/m^2^; and the exclusion criteria were: heart disease (including recent myocardial infarction, myocardial ischemia, and cardiac arrhythmia), chronic diseases of the liver, kidney, gastrointestinal system, musculoskeletal system, and central nervous system (CNS) as well as diabetes and dyslipidemia. The subjects were randomly divided into two groups of 11 (control and intervention groups). In the beginning of the eight-week intervention, spirometry was performed to both groups’ subjects; however, pulmonary volumes, including forced expiratory volume (FEV) 1, forced vital capacity (FVC), and FEV1/FVC ratio, were measured and recorded. After obtaining the informed consent for exercise with a full explanation of the exercises and the way of doing them, the aerobic exercises were set to fixed bikes and treadmills; the intervention was performed within eight weeks three 30–40-minute sessions per week; the duration of each session was oriented based on the patients’ stability. Before the intervention onset, the incremental test for each patient was performed to assess and determine the stability of patients to tolerate the exercise and prescribe the optimal exercise intensity. Finally, to assess the effectiveness of the exercises, the same test was performed. It should be noted that subjects who had the contraindications of exercise were excluded from the study.

During the implementation of the test, subjects reached the maximum activity criteria or symptom-limited activity. Electrocardiography (ECG), blood pressure (BP), heart rate (HR), and oxygen saturation (SaO_2_) as well as clinical manifestations were continuously controlled during the session for each patient and the intervention was discontinued in the event of fatigue or inability of the patient. In the case of SaO_2_ dropping to less than 88%, the oxygen was given to the patient and if the hypoxia was not corrected, the sports activity was discontinued and the subject was excluded from the study.

Blood samples were taken from both groups before and after the intervention, and Apo A-1 and Apo B levels were measured. Apo A-1 and Apo B levels and their ratio were measured and the before and after the intervention measures were compared within and between the groups.

Data collected from the demographic and clinical information checklist were transferred into the computer after encoding. Data were analyzed by descriptive tests including mean, SD, frequency and frequency percentage, and analytical tests including the paired samples *t* test and independent-samples *t* test. The Shapiro-Wilk test was used to assess the normality of the data that was not significant in the current test; therefore, the data normalization hypothesis was confirmed (P>0.05). In the current study, data were analyzed with SPSS version 20 and the significance level was considered <0.05.

## RESULTS

The mean FEV1 measured by spirometry in the aerobic exercise and control groups in the beginning of the study were 1.84 L (56.36 ± 20.66%) and 1.37 L (49.09 ± 12.06%), respectively.

According to the pulmonary function test and based on the GOLD classification used to classify COPD, in the aerobic exercise group, 27.27% of the patients were in the absence staining of the disease, 45.45% in the moderate staining, 18.18% in the intense, and 9.09% were in very intense staining. In the control group, the grading was 9.09%, 36.36%, 45.45%, and 9.09% in the absence, moderate, intense, and very intense staining of the disease, respectively. The characteristics listed in the two groups are shown in Table 1.

**Table 1.** Frequency distribution of demographic and clinical characteristics of patients, according to the two groups of aerobic and control exercises

**Variable**		**Intervention Group**		**Control Group**	

		Mean	SD	Mean	SD
Age		64.36	3.90	67.18	3.86
BMI		22.90	1.22	24.92	3.90
FEV1		1.84	0.70	1.37	0.31
**Variable**		N	%	N	%
Smoking	Yes	10	90.90	9	81.80
	No	1	9.10	2	18.20
COPD	Mild	3	27.27	1	9.09
	Moderate	5	45.45	4	36.36
	Sever	2	18.18	5	45.45
	Very Sever	1	9.09	1	9.09

The normal ranges of Apo A1 and Apo B were 110–190 and 75–155 mg/dL, respectively. Based on the results of the current study, in the aerobic exercise group, the means of Apo A1 before and after the intervention were 146 and 169.36 mg/dL, respectively. The results of the paired samples *t* test showed that the mean Apo A1 score in the aerobic exercise group increased after the intervention compared with that of before intervention, and the difference was statistically significant (P<0.05). In addition, the means of Apo B before and after intervention were 83.27 and 93.63 mg/dL, respectively. The results of the paired samples *t* test showed that the mean Apo B score in the aerobic exercise group after the intervention was significantly higher than that of before the intervention (P<0.05). However, in the aerobic exercise group, the mean ApoA1/Apo B ratio after the intervention (1.85) was not significantly different from that of before the intervention (1.80) (P>0.05).

**Figure 1.**
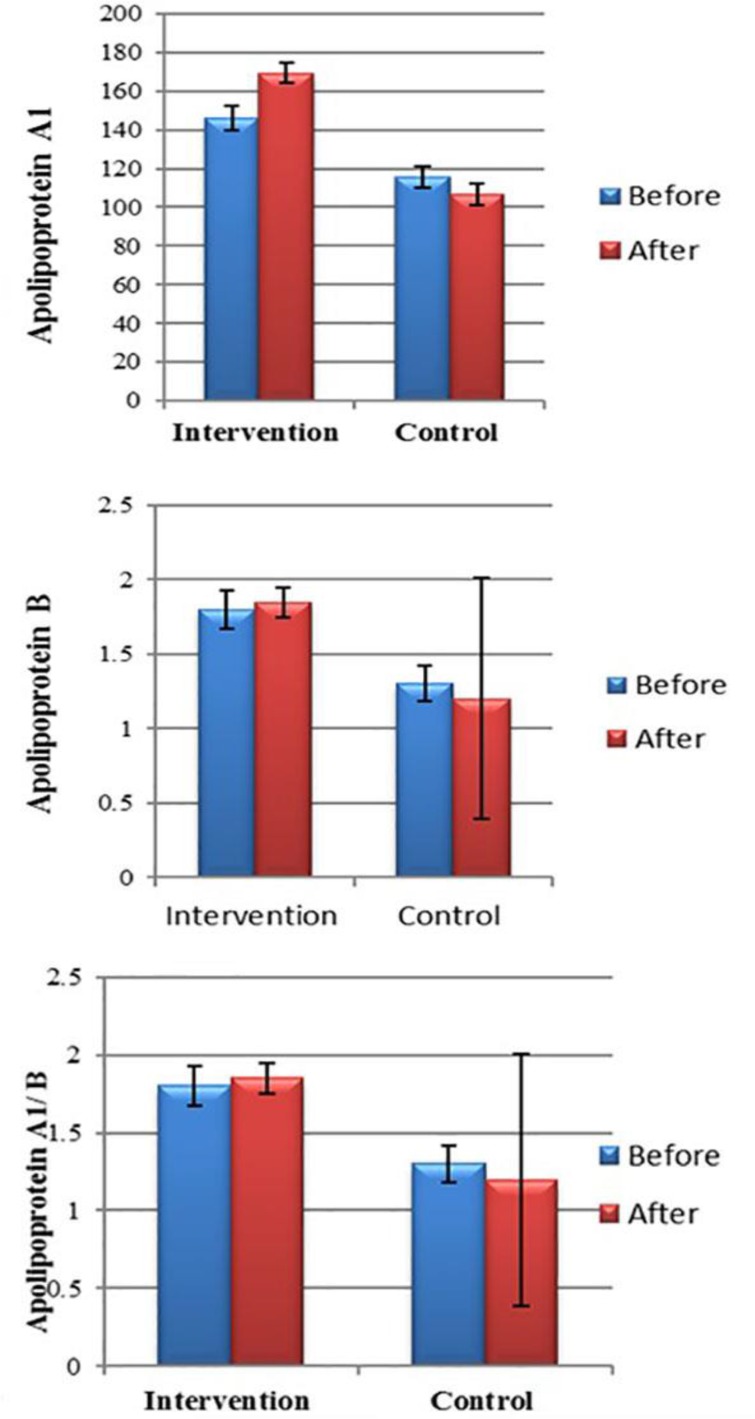
Distribution of mean serum levels and Apo A1 to Apo B ratio in patients, according to the two groups of aerobic and control exercises

In the control group, the means of Apo A1, Apo B, and Apo A1/Apo B ratio before the intervention were 115.45 and 98.90 mg/dL, and 1.30, respectively, which changed to 106.54 and 94.18 mg/dL, and 1.20 after the intervention, respectively. The results of the paired samples *t* test showed that the means of Apo A1 and Apo B, and Apo A1/Apo B ratio in the control group after the intervention were not significantly different from those of before the intervention (P <0.05) (Table 2) (Figure 1).

**Table 2.** The mean serum levels and Apo A1 to Apo B ratio in patients, before and after aerobic exercise in intervention and control groups

**Variable**	**Stage**	**Intervention Group**		**Control Group**	

		Mean	SD	Mean	SD
**Apolipoprotein A1**	Before Intervention	146.00	6.09	115.45	5.61
	After Intervention	169.36	5.42	106.54	5.40
	P-value	0.001^*^		0.15	
**Apolipoprotein B**	Before Intervention	83.27	4.44	98.90	12.80
	After Intervention	93.63	5.24	94.18	8.57
	P-value	<0.001^*^		0.63	
**Apolipoprotein A1/ B**	Before Intervention	1.80	0.13	1.30	0.12
	After Intervention	1.85	0.10	1.20	0.80
	P-value	0.53		0.21	

* P-values are significant.

In the current study, the intergroup analyses of ApoA1 and ApoB, and ApoA1/Apo B ratio changes showed that the mean of ApoA1 increased to 23.36 mg/dL in the intervention group, while decreased in the control group to 8.9 mg/dL and the difference between the groups in this regard was significant (P <0.05).

Also, ApoB in the intervention group increased by an average of 10.36 mg/dL and decreased in the control group with a mean of 4.72 mg/dL, and the difference between the measures were not statistically significant (P >0.05). The ApoA1/Apo B ratio in the intervention group increased by an average of 0.04 and decreased in the control group with a mean of 0.10, and the difference between the groups in this regard was statistically insignificant (P >0.05) (Table 3).

**Table 3.** Comparison of the average changes Apo A1, Apo B and A/B ratio in intervention and control groups

**Mean difference (after-before)**	**Intervention Group**		**Control Group**		**P-value**

	Mean	SD	Mean	SD	
**Apolipoprotein A1**	23.36	22.50	8.90-	22.27	0.004
**Apolipoprotein B**	10.36	12.73	4.72-	47.10	0.76
**Apolipoprotein A1/ B**	0.04	0.27	−0.10	0.44	0.13

## DISCUSSION

The results of studies conducted on large populations can play an important role in the management of COPD and further researches. In previous studies, it was observed that serum levels of tumor necrosis factor (TNF), endothelin 1, interleukin (IL)-6, and C-reactive protein (CRP) increased in patients with COPD compared with the healthy subjects.

Normal HDL level has a protective effect on the lung functioning in healthy people and the ones with asthma and lung cancer, which specifies its anti-oxidative and anti-inflammatory properties. As noted above, Apo A1is a structural protein of the HDL and has numerous protective effects on the healthy lung and pulmonary diseases(24). It was observed that ApoA1 and lipocalin-1 present in the sputum of patients with COPD significantly decreased compared with those of the healthy smokers (28). ApoA1 and Apo B were the better indicators of atherosclerotic disease than lipids and lipoproteins and Apo A1/Apo B ratio suggested a balance between these two and it was a more useful indicator of atherosclerotic diseases (29). Many studies were conducted to determine the role of different factors in the pathogenesis and the severity of pulmonary and cardiovascular diseases associated with them, aiming to create new therapeutic approaches to manage the diseases better. As noted, the protective role of ApoA1 in healthy lungs, particularly in the pathogenesis of various pulmonary and atherosclerotic diseases, is proven. ApoB has a proven role in atherosclerotic diseases of the cardiovascular system, but its role in the pathogenesis of pulmonary disease is not determined yet.

In the current study, a significant increase was observed in the mean serum Apo A1 level of post-intervention compared with pre-intervention in the aerobic exercise group and the significant changes in the aerobic exercise group compared with the control group showed that regular aerobic exercises can play an effective role in improving the respiratory efficacy in patients with COPD through an increase in the serum levels of ApoA1.

In the study by Basili et al., to determine the lipid profile and its relationship with vascular and heart diseases, level of Apo B, lipoprotein A, total cholesterol, HDL, LDL, and triglyceride levels were measured and compared with those of the healthy control population, and it was observed that patients with COPD did not have atherogenic lipid profiles and the increased risk of cardiovascular disease in such patients was not due to the effect of atherogenic lipid profile and its association with the disease (30).

It was also observed that the mean Apo B score significantly increased after the intervention compared with before intervention in the aerobic exercise group, but its average changes were not significant in comparison with those of the control group.

Finally, Apo A1/Apo B ratio in the aerobic exercise group insignificantly increased after the intervention compared with the pre-intervention, and its average changes were not significant in comparison with those of the control group; therefore, it could be argued that doing exercises in patients with COPD cannot prevent the predisposition of cardiovascular diseases through affecting lipid profiles.

However, the role of physical activities in reducing the risk of cardiovascular diseases is already determined; for example, a study found that higher levels of daily physical activity can reduce arterial stiffness and lead to decreased risk of cardiovascular events (31).

As noted above, there was no study on the role of Apo B in pulmonary diseases, and it is not apparent how its increase or decrease and the ratio of its change in favor of Apo A1 can affect the pathogenesis and progression of pulmonary diseases; therefore, it is suggested to investigate this topic in future researches.

In the current study, there was no correlation in Apo A1 and Apo B measures and Apo A1/Apo B ratio between the patients with COPD and the healthy controls in order to determine the impact and roles of such parameters in COPD; the issue that can be considered in future studies. Based on the protective role of Apo A1 in other types of pulmonary diseases, such as asthma, lung cancer, and pulmonary hypertension, a study on the effect of exercises on such diseases can also be valuable. Sports exercises in the current study were simple, cost-effective, and convenient; the results of the current study indicated the efficacy of exercises, in spite of the physical limitations of the patients with COPD in terms of doing exercises. In the beginning of the current study, it was expected that the lack of co-operation of patients with the authors to do aerobic exercises would be one of the limitations of the study, but given the positive role of these exercises in improving the physical and mental status of the patients, the cooperation of the study subjects with authors in terms of doing aerobic exercises was considered very good and did not make any specific limitations.

## CONCLUSION

Regular aerobic physical exercises are effective in increasing the serum level of Apo A1 in patients with COPD, and given the proven protective role of Apo A1 in patients with COPD, it can improve the respiratory efficacy of such patients. The exercises performed in the current study were very simple, cost-effective, and convenient and can be included in the pulmonary rehabilitation programs. Due to the fact that Apo A1/Apo B ratio did not significantly increase following the intervention, it is concluded that sports activities do not play a role in reducing the risk of cardiovascular diseases induced by atherogenesis in patients with COPD.
